# Fatal Donor-Derived Carbapenem-Resistant* Klebsiella pneumoniae* Infection in a Combined Kidney-Pancreas Transplantation

**DOI:** 10.1155/2016/7920951

**Published:** 2016-10-16

**Authors:** Giovanni Varotti, Ferdinando Dodi, Anna Marchese, Alessia Terulla, Massimo Bertocchi, Iris Fontana

**Affiliations:** ^1^Kidney Transplant Unit, IRCCS San Martino University Hospital, IST National Institute for Cancer Research, Genoa, Italy; ^2^Department of Infectious Disease, IRCCS San Martino University Hospital, IST National Institute for Cancer Research, Genoa, Italy; ^3^Department of Microbiology, IRCCS San Martino University Hospital, IST National Institute for Cancer Research, Genoa, Italy

## Abstract

Carbapenem-resistant* Klebsiella pneumoniae* (CR-KP) infections in solid organ transplant recipients are associated with high morbidity and mortality. We report a case of a fatal donor-derived CR-KP infection in a combined kidney-pancreas transplant. Given the short interval of time between donor hospitalization and organ procurement, information concerning the donor CR-KP positivity arrived only 72 hours after transplant. Based on this experience, we believe that knowledge of the donor's CR-KP status should be mandatory before procurement and, if positive, pancreas donation should be contraindicated.

## 1. Introduction

First described in 2001 concerning an isolate collected in a North Carolina Hospital in 1996, CR-KP infections have spread rapidly worldwide, becoming a serious healthcare problem associated with increased cost and length of stay as well as worse patient outcomes [1, 2]. Its impact can be particularly devastating in solid organ transplant recipients, as they are a very high-risk population because of either their immunosuppressed status or their prolonged hospitalization [3, 4].

Moreover, given their stay in intensive care units under mechanical ventilation and their severe illness requiring vasopressor support, deceased multiorgan donors are also another high-risk category for CR-KP transmission, leading potentially to donor-derived infection of recipients [5].

Unfortunately, definitive information concerning the donor's cultures may be still unknown during the times of procurement and implantation, resulting in an indefinite risk for donor-derived CR-KP transmission.

## 2. Case Report

We report the case of a 37-year-old diabetic female, who underwent a combined kidney-pancreas transplant in our centre in April 2014.

Donor was a 26-year-old male who remained in ICU for only 18 hours before procurement and was considered a “standard” risk for infections.

Simultaneous pancreas and left kidney transplant was performed by first placing the pancreatic graft in the right lower intraperitoneal cavity, with vascularization on the recipient's right common iliac axis and a duodenoenterostomy for the exocrine drainage. Then a standard kidney transplant was performed placing the graft in the left iliac fossa.

Intravenous (IV) amoxicillin doses were continued for 48 hours as perioperative antibiotic prophylaxis.

On POD 3, the donor's sputum, bronchoalveolar lavage cultures, and the rectal swab were positive for carbapenem-resistant* Klebsiella pneumoniae* (CR-KP), susceptible only to colistin (MIC = 2 mg/L); the donor's blood cultures were negative. Combined IV preemptive antibiotic therapy with colistin (loading dose: 9.000.000 IU, maintenance dose: 2.000.000 IU/BID), meropenem (dose: 2 gr./BID), and tigecycline (loading dose: 100 mg, maintenance dose: 50 mg/BID) was started.

Four days later, the recipient's blood cultures revealed a CR-KP infection and a picture of peritonitis required an urgent relaparotomy which showed an enteric leak from duodenoileal anastomosis with surrounding necrotic tissue, which imposed the removal of the pancreatic graft.

Evidence of CR-KP was confirmed in the intraoperative swabs collected from the duodenal graft.

Comparison of all the donor's isolates to the recipient's blood isolate by pulsed-field gel electrophoresis [1] revealed an identical profile, confirming the donor-derived infection. The identifications of the KPC-3 gene were achieved by PCR and sequencing using primers and conditions previously described [6]. Genomic DNA was prepared, digested with* Xba*I (New England Biolabs Inc., MA, USA) and subjected to PFGE with the CHEF DRII device (Bio-Rad, Milan, Italy). All isolates exhibited the same PFGE macrorestriction profile, confirming the donor-derived infections.

Antibiotic was continued for six weeks, until cultures tested negative; on POD 70, the patient was discharged with normal kidney function, remaining colonized for CR-KP by positive rectal swab.

One month later, the patient was readmitted for a pseudoaneurysm of the right common iliac artery with surrounding liquid collection, found to be CR-KP positive and colistin resistant (MIC = 3 mg/L, EUCAST confirmed). Patient underwent right iliac replacement with heterologous homograft which was then infected by CR-KP requiring a subsequent silver graft substitution. Triple IV antibiotic therapy with ertapenem (500 mg/daily), meropenem (2 g/BID), and tigecycline (loading dose: 100 mg, maintenance dose: 50 mg/BID) was reintroduced but the iliac wound cultures remained CR-KP positive, subsequently developing a picture of sepsis. In the following weeks, the patient's condition progressively worsened and, unfortunately, she died as a result of multiorgan failure 195 days after transplant.

## 3. Discussion

There is very little data on this argument and, to the best of our knowledge, this is the first case reporting a donor-derived CR-KP infection in a kidney-pancreas transplant recipient. In the 15 published cases of donor-derived CR-KP infection in solid organ transplant (5 kidneys, 5 livers, 3 lungs, 1 combined liver-kidney, and 1 heart), 13 had an uneventful outcome while 2 recipients died (one after receiving a lung from an asymptomatic carrier of CR-KP in the respiratory tract and one after receiving a kidney from a donor with CR-KP urine infection) [3–5, 7, 8]. These initial experiences suggest that, in well-defined conditions and following a strict follow-up, organs from colonized CR-KP donors may be considered for transplantation, avoiding their use when the donor's blood cultures were positive or when the organ is directly involved in CR-KP infection, such as lungs in the case of the airway infection or kidneys in urinary tract isolation [7, 8].

However, our experience revealed peculiar aspects of pancreas donation. In a colonized subject, the CR-KP bacteria remain silent and mainly at the level of the gastrointestinal tract; in this setting, in the event of a pancreas transplant in which the graft necessarily includes a duodenal tract, there could be a high potential risk of direct bacterial transmission from the duodenal graft to the recipient.

In our case, the intraoperative swab collected proved the presence of CR-KP bacteria in the explanted duodenal graft. However, we cannot state that the CR-KP infection was directly responsible for the duodenal-ileal anastomotic leak but it was certainly one of the main causes of the sequence of unfavourable events which led to the patient's death. As a result of this experience, we believe that pancreas donation should also be contraindicated in the event of colonized CR-KP donors.

Given the short time interval between donor hospitalization and organ procurement (<24 hours), we learnt of the positivity of the rectal swab only three days after transplant. We believe it would be crucial to receive this information before harvesting, to optimize the organ allocation process. These reasons highlight the importance of prompt interinstitutional information sharing among the donor's hospital doctors, local organ coordination centre, and transplant teams. Moreover, we believe that in selected cases, such as ours, the use of faster diagnostic assays such as real-time PCR [9, 10] should be encouraged for rapid CR-KP detection.

## Abbreviations

CR-KP: Carbapenem-resistant* Klebsiella pneumoniae*
ICU: Intensive care units.
